# Hepatitis Delta Virus: A Peculiar Virus

**DOI:** 10.1155/2013/560105

**Published:** 2013-10-02

**Authors:** Carolina Alves, Cristina Branco, Celso Cunha

**Affiliations:** Medical Microbiology Unit, Center for Malaria and Tropical Diseases, Institute of Hygiene and Tropical Medicine, Nova University, Rua da Junqueira 100, 1349-008 Lisbon, Portugal

## Abstract

The hepatitis delta virus (HDV) is distributed worldwide and related to the most severe form of viral hepatitis. HDV is a satellite RNA virus dependent on hepatitis B surface antigens to assemble its envelope and thus form new virions and propagate infection. HDV has a small 1.7 Kb genome making it the smallest known human virus. This deceivingly simple virus has unique biological features and many aspects of its life cycle remain elusive. The present review endeavors to gather the available information on HDV epidemiology and clinical features as well as HDV biology.

## 1. Introduction

In 1977, a novel antigen was found in the nucleus of hepatocytes from patients with a more severe form of hepatitis B. It was first thought to be a previously unknown marker of hepatitis B virus (HBV). Only later, it was found that the then called delta antigen was not part of HBV but of a separate defective virus that requires the presence of HBV for infection. The newfound virus was designated hepatitis delta virus (HDV) and, by 1986, its RNA genome was cloned and sequenced (reviewed by [1]). This peculiar virus has been classified as the only member of the genus *Deltavirus* due to its uniqueness [2]. The HDV virion is a hybrid particle, composed of the delta antigen and HDV RNA enclosed by the surface antigens of HBV (HBsAgs). HDV has the smallest RNA genome of all known animal viruses. However, it is comparable, although larger, to viroid RNAs, pathogenic agents of higher plants.

## 2. Epidemiology

HDV infection is distributed worldwide, although not uniformly, and it is estimated that 5% of HBsAgs carriers are also infected with HDV, which signifies that there might be between 15 and 20 million HDV-infected individuals [3]. This is a very rough number because it lacks data from areas where HBV is highly prevalent and HDV is poorly studied.

HDV is highly endemic in Mediterranean countries, the Middle East, northern parts of South America, and Central Africa [4]. HDV also has high prevalence in Turkey [5], Central Asia [6], and the Amazonian region of Western Brazil [7].

In Southern Europe, HDV infection has been highly prevalent, with studies from the 1980s and 1990s showing that the incidence of HDV in HBsAgs positive individuals was higher than 20% [8]. With the implementation of HBV vaccination programs in the 1980s, HDV prevalence considerably decreased to 5–10% by the late 1990s [9]. However, in the beginning of the XXI century, the number of HDV-infected HBsAgs carriers in Europe increased to 8–12% [9, 10]. This increase has been attributed to immigration of individuals from highly endemic regions [10]. Another report claims that the increase in HDV incidence is not only due to immigration but also due to other factors associated with HDV modes of transmission [9]. Drug addiction and other risk behaviors, such as multiple sexual partners, tattooing and piercing, or uncontrolled medical procedures, have been shown to contribute to the spread of hepatitis D in Italy [9]. In fact, in western countries, the virus is highly prevalent in intravenous drug addicts with chronic HBV infection [9, 10].

More recent and reliable data are needed, especially from poorly studied regions. As an example, only recently are data starting to emerge from the United States of America. A 2013 survey has shown that in Northern California, 8% of 499 chronic HBV patients tested positive for HDV infection [11].

Based on nucleotide sequence analysis, eight HDV genotypes have been defined, some of which are distributed by distinct geographic regions [4, 12]. The divergence in nucleotide sequence between isolates of the same genotype is less than 15% and between different genotypes it can be as high as 40% [4]. HDV genotype 1 is the most common and prevalent worldwide, present mainly in Europe, Middle East, North America, and Northern Africa. It is associated with both severe and mild forms of the disease [13]. Genotype 2 is more common in the Far East, present in Japan, Taiwan, and parts of Russia [14]. Genotype 2 is associated with a milder disease course [13]. HDV genotype 3 is exclusively found in the Amazon Basin [7] and has been associated with the most aggressive forms of HDV infection. The combined infection of HDV genotype 3 and HBV genotype F was associated with fulminant hepatitis in South America [15]. Genotype 4, present in Japan and Taiwan [16], has variable pathogenicity. A genotype 4 isolate from Okinawa, Japan, has been associated with greater progression to cirrhosis than the genotype 4 predominant in Taiwan [17]. Genotypes 5 to 8 were found in African patients who had migrated to Northern Europe [4, 12]. Phylogenetic reconstructions based on the delta antigen coding sequence have shown a probable ancient radiation of African lineages [4].

## 3. Clinical Expression

Hepatitis delta virus is usually associated with a severe form of hepatitis, but the range of clinical manifestations is very wide going from asymptomatic cases to fulminant hepatitis.

Regarding HDV transmission, like its helper virus HBV, it is parenterally transmitted through exposure to infected blood or body fluids. Intrafamilial spread is naturally common in highly endemic regions.

HDV requires the presence of HBsAgs to form new infectious virions and propagate HDV infection. Thus, hepatitis D only occurs in individuals infected with HBV. Consequently, there are two major patterns of infection: “coinfection” with HBV and HDV or “superinfection” of patients already infected with HBV. A rare third pattern has been reported; it can occur after liver transplantation for an HDV-infected individual and is designated as “helper-independent latent infection” [18]. In this scenario, an initial HDV infection of the new liver occurs without any apparent help from HBV. Such an infection remains asymptomatic unless reactivated by HBV appearance [18].

For an HBV and HDV acute coinfection, the most common outcome (95%) is viral clearance [14]. However, it can be more severe than an acute HBV monoinfection, resulting in some cases in acute liver failure [19]. Acute hepatitis strikes after an incubation period of 3–7 weeks, beginning with a period of nonspecific symptoms such as fatigue, lethargy, or nausea [20].

HDV superinfection of chronic HBV patients also causes severe acute hepatitis, but in this case, for up to 80% of patients, it progresses to chronicity [21]. The processes, which determine whether a patient clears HDV spontaneously or becomes chronically infected, remain unclear. When chronic HDV infection is established, the preexisting liver disease caused by HBV is usually aggravated [22]. It has been claimed that, during the acute phase of HDV infection, HBV replication is suppressed to very low levels and that this suppression can persist once a chronic HDV infection is established [23]. Patients with HDV superinfection suffer a more rapid progression to cirrhosis [24, 25], increased liver decompensation, and eventually death [26], when compared with patients with HBV monoinfection. Despite the higher rates of progression to cirrhosis, not all published studies refer to an increased rate of hepatocellular carcinoma [27]. One explanation of this may be the abovementioned suppression of HBV replication by HDV, since other studies assert that higher HBV DNA serum levels correlate with a greater risk of carcinoma [28].

## 4. Diagnosis

Since HDV is a satellite virus of HBV, every HBsAgs positive patient should be screened for coinfection with HDV; that is, patients should be tested, at least once, for anti-HDV antibodies. A negative result does not justify testing for HDV RNA as, so far, it seems that every individual infected with HDV develops anti-HDV antibodies [29]. In contrast, a positive result for anti-HDV antibodies requires confirmation of continued HDV infection, through detection of HDV RNA in serum. Anti-HDV antibodies may be present even after HDV RNA has disappeared during recovery from the infection [29].

Currently, there is no need for quantification of the HDV RNA levels in serum during the diagnosis step. There is no evidence that a correlation exists between the stage of liver disease and the levels of HDV RNA [30]. Thus, a liver biopsy is still the major tool for evaluating the stage of delta hepatitis in patients [29]. However, a quantitative assay of HDV RNA is useful during the therapy stage to monitor the treatment response of patients undergoing therapy. Unfortunately, very few data are available on the levels of HDV RNA during the different stages of the disease. Thus, there is no accepted threshold level at which one might recommend treatment.

For some time, quantification of HDV RNA levels in clinical samples has suffered from the lack of a standardized test. Quantification of HDV RNA was done in specialized laboratories using in-house protocols, which unfortunately become irrelevant outside the laboratory of origin. Such assays typically lacked an internal control and were limited to only one genotype. Furthermore, there is no international reference standard to make results from different laboratories comparable. As proposed elsewhere, an HDV RNA reference preparation should be defined by the World Health Organization to be used as an international standard [31].

In 2012, two standard protocols were proposed to detect and quantify HDV RNA from clinical samples [32, 33]. One method is described as able to be automated to accurately quantify the major HDV genotypes present in Europe (genotype 1 and the migrant African strains 5–8; [32]). The other standardized test is described as being able to detect and quantify all HDV genotypes [33]. Both protocols use a commercial kit to extract nucleic acids from samples and include an internal control to enable monitoring of the overall performances of the assay. The one-step RT-qPCR makes use of another commercial kit [32, 33].

Application of standardized procedures is crucial to improve our understanding of HDV RNA kinetics during the course of disease. It will improve patient management, as data can be gathered which will help in the decision to start treatment, as well as monitoring the response to therapy in chronic patients. Also it will contribute to the screening of HDV infections in the endemic areas, providing more reliable epidemiological data. Overall, acceptance of standardization will help clarify the pathophysiology of HDV infections.

## 5. Treatment

Ideally, a successful treatment of an HDV infection eradicates HDV and its helper virus HBV. Clearance of HDV is obtained when both HDV RNA and HDAg in the liver become persistently undetectable and a complete resolution is achieved when HBsAgs clearance is also obtained.

However, at this time, there is no efficient therapy. Prolonged treatment with recombinant interferons is the only therapy that has shown antiviral activity against HDV. Such therapies, which last up to 2 years, have been reported as only 20–40% efficient [34].

In general, when searching for a treatment for viral disorders, the first and preferred targets analyzed are the viral components, such as enzymes involved in the virus replication cycle. But HDV lacks any specific enzymatic function to target. Since the only known enzymatic activity the virus possesses is a ribozyme, the virus relies on the host cell to provide for all other enzymatic activities needed for its life cycle. This represents a serious challenge in finding an HDV-specific therapeutic target.

Puzzlingly, the nucleoside and nucleotide analogues used for treatment of HBV infection are inefficient against HDV. Although they block HBV DNA synthesis in chronic patients, they have little impact on HDV and do not even enhance interferon treatments [34]. Famciclovir, lamivudine, and adefovir, all used in HBV treatment, have been shown to lack any significant antiviral activity against HDV [35–37]. Ribavirin, a nucleotide analogue, which inhibits HDV replication in cell culture, when administered alone or in combination with interferon, also failed to increase rates of HDV RNA clearance [38].

Interferon-*α* (IFN-*α*) has been used for treatment of HDV infections since the mid-1980s [39]. Several trials were carried out exploring different doses and durations. Responses to treatment varied and clearance occurred at different times from the beginning of treatment, occurring even after discontinuation of treatment [35]. Researchers have yet to identify pretreatment characteristics that determine responders and nonresponders to IFN-*α* therapy. It seems that 2 years of treatment with IFN-*α* is superior to shorter treatment durations to obtain HDV RNA clearance [35]. It has been reported that in a 1-year treatment, there is only a 10 to 20% chance of HDV clearance, and in a 2-year treatment trial, 20% of patients were cleared [40]. The rate of response is proportional to the dose of IFN-*α*; patients treated with doses of 9 million units responded better than those treated with only 3 million units, and relapse was common when the IFN-*α* dose was reduced [35]. Unfortunately, a prolonged treatment with high doses of IFN-*α* is tolerated by only a minority of patients [29]. IFN-*α* side effects include flu-like symptoms, fatigue, and weight loss as well as severe psychiatric disturbances. Patients have a tendency to become deeply depressed; suicides and attempted suicides have been reported [35]. The severity of reactions tends to be proportional to IFN-*α* dose, and intermittent use of IFN-*α*, observed in drug abusers, increased incidence and severity of side effects [35].

By 2006, IFN-*α* was largely replaced by longer-lasting pegylated IFN-*α* (PEG-IFN-*α*) [38, 41, 42]. Clearance of HDV RNA was obtained for 6 out of 14 patients in a 1-year treatment plan [41]. However, in a similar study, only 2 patients in 12 were cured [42]. In a third study, 8 patients out of 38 became HDV RNA negative after 72 weeks of treatment [38]. Ribavirin was also used in this trial but without any apparent beneficial effect [38].

The Hep-Net International hepatitis D intervention trial, which included 90 patients from Germany, Greece, and Turkey, tested PEG-IFN-*α*2a alone or with adefovir and adefovir alone [36]. HDV RNA clearance was only observed in patients who had received treatment including PEG-IFN-*α*2a, showing an antiviral efficacy in more than 40% of patients, and 25% became HDV RNA negative [36]. Adefovir showed little efficacy in reducing HDV RNA levels, but a PEG-IFN-*α*2a plus adefovir therapy was superior in reducing HBsAgs serum levels [36].

Currently, it is usually recommended to treat chronic hepatitis D with PEG-IFN-*α* for one year or longer, if the patient can tolerate the adverse effects of such therapy [14]. For patients with advanced liver disease, liver transplantation is the only therapy available [40].

An optimization of the available treatment strategies is clearly needed, either regarding doses or duration, and also possible combinations such as PEG-IFN-*α*2a with adefovir to also tackle HBsAgs, crucial for HDV propagation. Most importantly, alternative treatments need to be explored, as the efficacy of the current therapies is clearly unsatisfactory. One of the most promising alternatives is the prenylation inhibitors since, as will be discussed subsequently, prenylation of HDAg is essential for interaction with HBsAgs. Furthermore, prenylation inhibitors have already been developed to treat a number of malignancies and were shown to be safe [43].

## 6. HDV Biology

### 6.1. HDV Virions and Putative Host Cell Receptors

An infectious HDV virion is an enveloped, roughly spherical particle, of around 36 nm in diameter [44]. The outer coat of the virion containing host lipids and the HBsAgs surrounds an inner nucleocapsid consisting of viral ribonucleoproteins (RNPs) with the genomic RNA and about 200 molecules of HDAg per genome [45].

Since HDV and HBV share the same envelope proteins, it is often assumed that attachment and cell entry occur via similar mechanisms. 

Several studies have attempted to identify the regions of the HBsAgs required for HDV and HBV entry. The preS1 region of L-HBsAg is myristoylated at the N-terminus. This posttranslational modification and about 48 adjacent amino acids are essential for HBV and HDV entry into hepatocytes. Synthetic peptides that mimic this region are potent inhibitors of virus entry [46].

Many studies have aimed to discover the host receptors for HBV (and maybe HDV). Many candidates have been proposed but not confirmed [47].

It has been suggested that functional purinergic receptors are required for HDV entry as compounds that block the activation of such receptors inhibited HDV and HBV infection of primary human hepatocytes [48]. However, a different study has since reported that such blocking compounds interfere in HDV and HBV infection due to their charge and not because the receptors are directly involved in the process [49]. In fact, one of the blocking compounds used, ivermectin, reduced HDV infection when added after virus inoculation just as well as when added before inoculation [49]. This suggests that ivermectin focuses on a step of the HDV replication cycle other than the receptor binding stage. In this study, it has also been shown that HDV cell entry depends on binding to the glycosaminoglycan side chains of the hepatocyte heparan sulfate proteoglycans, much like what has been observed for HBV infection [49].

In contrast to all previous studies, an important new report by Yan and colleagues demonstrates that a necessary and sufficient receptor for HBV and HDV is the sodium taurocholate cotransporting polypeptide [50]. This protein is a multiple transmembrane transporter expressed in the liver. Silencing expression of this protein in primary hepatocytes using small interfering RNAs inhibited HBV and HDV infection. Expression of this protein in human liver cell lines rendered them susceptible to infection by HBV and HDV. Therefore, it is now possible for the first time to study the infection processes for these viruses *in vitro*, using established human liver cell lines, which are much more convenient and reproducible than primary hepatocyte cultures.

### 6.2. HDV RNAs

HDV has a small circular RNA genome with only ~1700 nucleotides; this sequence length varies by no more than 30 nucleotides among HDV isolates [51]. In native conditions, the RNA folds into an unbranched rod-like structure due to intramolecular base pairing involving around 74% of its nucleotides [52].

HDV contains one functional open reading frame (ORF), encoding the delta antigen [53]. This ORF is not encoded by the genomic RNA but by another RNA species that arises during replication, the HDV antigenome, an exact complement of the genome.

**Figure 1:**
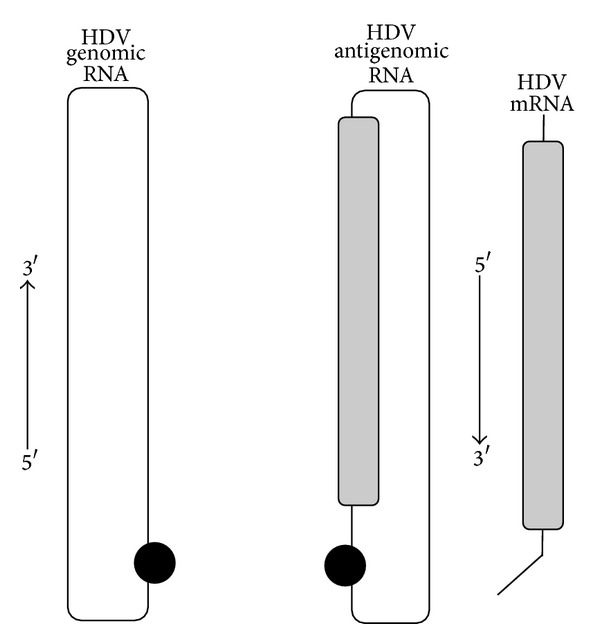
Schematic representation of three HDV RNA species. The HDV genomic RNA is a single-stranded circular RNA with ~1700 nucleotides. It forms an unbranched rod-like structure due to intramolecular base pairing. The HDV antigenomic RNA is the exact complement of the genomic RNA, and both RNAs have site-specific ribozymes indicated by the black circle. The HDV antigenomic RNA contains the open reading frame for the delta antigen, represented in grey, but the antigen is translated from another RNA species, the mRNA. The mRNA is ~800 nucleotides long with a 5′-cap and a 3′-polyadenylated tail.

The delta antigen is translated from a third RNA species, a linear 0.8 Kb messenger RNA (mRNA) of antigenomic polarity and a 5′-cap and 3′-polyadenylated tail [54]. The different HDV RNA species are represented in Figure 1. In an infected cell, the three HDV RNA species accumulate in very different amounts, although genomic RNA is the only species assembled into HDV virions. HDV genomic RNA is the most abundant; around 300,000 copies accumulate in an infected cell whereas 100,000 copies of the antigenome are present [53]. The HDV mRNA is considerably less abundant with approximately 500 copies per cell [55].

Site-specific self-cleavage and ligation has been reported on antigenomic HDV RNA, showing that this RNA possesses ribozyme activity, just like plant viroids [56]. Both genomic and antigenomic RNAs display this ribozyme activity, which is comprised within a contiguous sequence of less than 100 nucleotides [57]. They enhance HDV RNA self-cleavage by a 10^6^- to 10^7^-fold when compared with uncatalyzed cleavage [58, 59]. Although ribozymes are characteristic of viroids, their structures are different from HDV ribozymes, which are actually more related to the cytoplasmic polyadenylation element-binding protein 3 (CPEB_3_) ribozyme, a conserved mammalian sequence within an intron of the CPEB_3_ gene [60]. In fact, numerous HDV-like ribozymes have since been found in several eukaryotic species [61].

### 6.3. HDV RNA Replication

**Figure 2:**
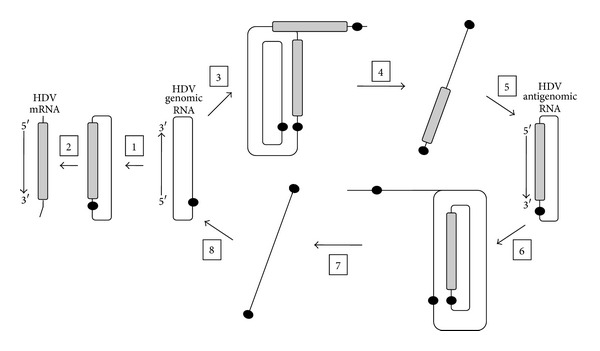
Model of HDV replication through a rolling-circle mechanism. The HDV genomic RNA is used as a template for the precursors of HDV mRNA (Steps 1-2) and also acts as a template for multimeric RNAs of antigenomic polarity (Step 3). These multimeric RNAs contain at least two copies of the HDV ribozyme and are thus self-cleaved to produce linear unit-length HDV antigenomes (Step 4), which are then ligated to produce circular antigenomic RNA (Step  5). In turn, the new antigenomic RNA is a template for multimeric RNAs of genomic polarity (Step  6) that are similarly self-cleaved and subsequently ligated to produce new circular genomic RNA (Steps  7-8).

HDV RNAs are transcribed in the nucleus of infected cells, but the details of this process remain poorly defined. The three RNA species that accumulate in infected cells are the product of posttranscriptional processing. The precursors, from which they arise, are thought to be transcribed by a double-rolling circle mechanism, exemplified in Figure 2. In this model, the circular genome RNA is used as a template to produce multimeric species of opposite polarity [62]. These greater than unit-length RNAs are subsequently self-cleaved by the HDV ribozymes and religated, producing unit-length circular antigenomic RNAs. The religation step is thought to involve a host ligase [63] although it has been shown that the HDV ribozyme can self-ligate *in vitro* [64]. Through a similar mechanism, the unit-length circular antigenomic RNA acts as a template for the transcription of multimeric species, which are processed to produce genomic RNA. The genomic RNA also acts as a template for transcripts that are processed into mRNA.

Even though such a rolling-circle mechanism has been widely accepted as a model for HDV replication, critical details remain to be confirmed and/or clarified such as the host cell components involved (reviewed by [65]).

HDV has no known DNA intermediate, as observed for retroviruses [65], and the only HDV protein, the delta antigen, is too small to be a polymerase. This means that HDV RNA must somehow redirect host DNA-dependent RNA polymerases to use HDV RNAs as templates. How this is achieved and which host polymerase(s) is (are) involved have been extensively studied but the results remain somewhat controversial.

The host RNA polymerase II (pol II) seems to be required for genomic HDV RNA transcription. Nuclear run-on experiments on an endogenous HDV RNA template have shown that inhibition of pol II by low concentrations of the specific inhibitor *α*-amanitin blocks HDV RNA synthesis of both the genomic and antigenomic strands [66]. One possible explanation is that the rod-like conformation of HDV RNAs may trick pol II into accepting the RNA as a double-stranded DNA template. It has been shown, through immunoprecipitation assays, that pol II binds the terminal stem loop regions of HDV genome [67]. It has also been reported that, after binding to the stem-loop, pol II is able to elongate multimeric RNA species, carrying out transcription [68]. Such elongation was observed on a partial antigenomic RNA stem loop and originated a chimeric molecule of newly synthesized transcript covalently bound to the 5′-end of the template. Thus, it is not clear if such elongation is biologically relevant.

Despite being shown that pol II interacts with genomic HDV RNA, it has been suggested that a different host polymerase is responsible for the synthesis of antigenomic HDV RNA [69, 70]. The idea that at least two different host polymerases are involved in the HDV replication cycle is based on the observation that, in transfected cells, the synthesis of new HDV antigenomic RNA was not inhibited by concentrations of *α*-amanitin that would inhibit pol II activity [70]. This has led to the speculation that pol I copies genomic HDV RNA to produce new antigenomic RNA [70]. This is contrary to the aforementioned nuclear run-on assays, which have shown that both genomic and antigenomic RNA syntheses are sensitive to low doses of *α*-amanitin, consistent with pol II involvement [66]. Note that HDV RNA has been detected in the nucleoplasm of cultured cells with nucleolus exclusion [71]. This suggests that if another host polymerase, other than pol II, is involved in HDV RNA replication, it is pol III, rather than pol I, which is resistant to high concentrations of *α*-amanitin.

An additional complication arises from *in vitro* studies, which indicate that fragments of the HDV RNA genome interact not only with pol II but also with pol I and pol III [72]. However, such *in vitro* interactions may not have biological relevance, especially since they do not lead to RNA-directed transcription.

The HDV mRNA possesses characteristics of a pol II transcript that is processed to a mRNA, namely, a 5′-cap structure and a 3′-poly(A) tail. In fact, the role of pol II in HDV mRNA transcription has been generally accepted [66, 69, 70, 73, 74].

The controversy regarding the transcription process is thus limited to whether genomic RNA is transcribed by pol II or another polymerase, either pol I or pol II [75]. If different polymerases are involved, then distinct metabolic requirements, as well as accessory factors, are necessary to accomplish these processes (reviewed in [76]).

In addition to the posttranscriptional processing to make the abovementioned three HDV RNAs, there is an important RNA-editing event. During the virus replication cycle, some of the antigenomes are edited at a specific site by a host adenosine deaminase (ADAR1). This changes the adenosine in the amber codon to inosine. After subsequent RNA-directed RNA synthesis, it leads to the replacement of inosine with guanosine [77]. That is, the UAG stop codon is changed to a UGG tryptophan codon. In this way, the delta antigen ORF is extended by 19 amino acids, that is, to the next stop codon. The specificity of the editing site is in part directed by the specific folding of the HDV antigenomic RNA [78].

Therefore, although HDV has only one ORF, it encodes two proteins: the small delta antigen (S-HDAg) of 195 amino acids and the large delta antigen (L-HDAg) with 214 amino acids.

## 7. Delta Antigens

**Figure 3:**
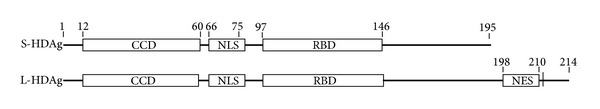
Functional domains of S-HDAg and L-HDAg. The delta antigens share most of their sequence differing only in the 19-amino-acid extension at the C-terminal of L-HDAg. They have, within the common sequence, as represented, a coiled-coil domain (CCD), a nuclear localization signal (NLS), and an RNA binding domain (RBD). Also indicated on L-HDAg are the nuclear export signal (NES) and the unique cysteine, residue 211, which is the target for farnesylation. The numbers indicate the position of the amino acid residues.

The two delta antigen isoforms share 195 amino acids and differ only in that the large form has 19 extra amino acids on the C-terminus. As such, S-HDAg and L-HDAg share several functional domains within the common amino acid sequence, as illustrated in Figure 3. The delta antigens contain a nuclear localization signal (NLS) comprised by amino acids 66 through to 75 [79]; a coiled-coil domain (CCD), also referred to as dimerization domain, within amino acids 12 to 60; and an RNA binding domain within amino acids 97 and 146 [80]. L-HDAg has, within its extra sequence, a nuclear export signal (NES) spanning amino acids 198 to 210 [81].

Both delta antigens undergo posttranslational modifications (PTMs) by several host enzymes. Several groups have investigated the impact these PTMs may have on the antigens' functions, but the precise significance of most of these modifications remains uncertain.

The exception is one PTM, characteristic only of L-HDAg, which has been shown to be essential. It occurs on cysteine residue 211 and is mediated by a host farnesyltransferase [82, 83]. This isoprenylation of L-HDAg is necessary, although not sufficient for viral packaging. It is somehow necessary for the interactions with HBsAgs, leading to the assembly of new viral particles [84, 85].

There are other PTM events, ones shared by both forms of the delta antigen. These involve phosphorylation, methylation, acetylation, and sumoylation (reviewed in [75]).

Phosphorylation has been observed at multiple sites, mostly at serine and threonine residues. Different phosphorylation patterns were observed for S-HDAg and L-HDAg, and, if relevant, the distinct patterns may in part account for their distinct biological functions [86]. Several host enzymes have been reported to phosphorylate delta antigens at different sites: casein kinase II on Ser2 and Ser213 [87]; double-stranded RNA-activated protein kinase R on residues Ser177, Ser180, and Thr182 [88]; extracellular signal-related kinases 1 and 2 (ERK1/2) on Ser177 [89]; and protein kinase C on residue Ser210 [87]. It has been alleged that S-HDAg phosphorylation increases replication of genomic HDV RNA from the antigenomic strand [89]. By enhancing the expression of ERK1/2 in cells transfected with plasmids expressing S-HDAg and dimeric HDV antigenomic RNA, an increase in the accumulation of HDV genomic RNA was observed but not for antigenomic RNA [89]. More recently, it has been suggested that phosphorylation of S-HDAg at Ser177 can work as a switch in HDV antigenomic RNA replication from the initiation to the elongation stage [90].

Acetylation of Lys72 on S-HDAg, by host p300 acetyltransferase, is thought to regulate nucleocytoplasmic shuttling of viral RNA [91, 92]. Note that this amino acid is within the NLS of the HDAgs [79]. Thus such a modification could be expected to have an impact on nuclear import. Acetylation of S-HDAg has also been suggested to function as a switch in the synthesis of the different viral RNA species as this PTM was reported to be essential for HDV genome and mRNA synthesis but dispensable for antigenomic RNA synthesis [93].

Methylation of Arg13 on S-HDAg, by protein arginine methyltransferase I, has been observed *in vitro* and has also been proposed to have a switching effect on HDV RNA replication [93, 94]. The studies were performed with S-HDAg with an R13A mutation, which failed to be methylated *in vitro*. In transfected cells, the mutant S-HDAg reduced genomic RNA synthesis and almost completely suppressed HDV mRNA synthesis [93, 94].

Finally, SUMOylation of multiple lysine sites, by small ubiquitin-related modifier isoform 1 (SUMO1), has been reported. Such PTM was detected on S-HDAg but not on L-HDAg [95]. And this PTM was proposed to enhance genomic RNA and mRNA synthesis based on experiments where SUMO1 was fused to S-HDAg, so as to mimic SUMOylated S-HDAg [95].

Although the two delta antigens share sequence and functional domains, they play very distinct roles in the HDV replication cycle. S-HDAg is essential for HDV RNA accumulation, whereas L-HDAg acts as a dominant negative inhibitor of HDV replication [96] and also is essential for the assembly, via HBsAgs, of HDV RNA into new virus particles. There is, however, a common function attributed to both antigens: it has been observed that both can downregulate HBV replication in cultured cells [97].

Regarding the appearance of L-HDAg, it is important to recall that because of the accumulated editing of the HDV antigenome, the proportion of this form, in relation to the total amount of accumulated HDAgs, increases during the replication cycle from 0% to around 30%, [98]. This is sufficient to suppress replication but allows the accumulation of viral genomes that can then be packaged into new infectious particles with the help of L-HDAg. The NES present in L-HDAg allows the viral RNP to be exported from the nucleus to the cytoplasm for packaging [81]. HDV RNPs then interact with HBsAgs at the endoplasmatic reticulum to form new infectious virions, which are then secreted to propagate further rounds of HDV infection [71]. Such assembly of new virions only occurs when HBsAgs are present; otherwise, the viral RNPs return to the nucleus [71].

S-HDAg has been more thoroughly studied than the large form, likely due to the fact that it is required for the accumulation of HDV RNA. Several roles have been attributed to this viral protein including putative and observed functions.

S-HDAg is present in the virions forming viral RNPs with the HDV genome. One of the first tasks it performs is the transport of the viral genome into the nucleus of infected cells, where RNA-directed RNA synthesis takes place. This transport is achieved by the presence of the previously described NLS and RBD. Nuclear import may be facilitated by karyopherin 2*α*, since this importin interacts with S-HDAg *in vitro* [99].

Another role attributed to S-HDAg is the regulation of HDV RNA editing, particularly the deamination by ADAR-1. This editing seems to occur at multiple locations on HDV RNAs, but it is focused on the antigenomic RNA at the stop codon adenosine [100]. S-HDAg has been found to suppress editing at this stop codon when expressed in transfected cells at levels close to those observed during HDV replication [100]. This observation suggests that the antigen plays a role in limiting HDV RNA editing, as excessive editing has been shown to inhibit HDV RNA accumulation [101]. 

It has been known for more than two decades that the small form of the delta antigen is essential for the accumulation of processed HDV RNAs [96]. Several theories have been proposed for the precise role(s) it may play, as will be discussed ahead. 

S-HDAg has been shown to interact with host pol II. In a pull-down assay, both S-HDAg and L-HDAg fused with a glutathione S-transferase tag were able to bind pol II from HeLa nuclear extracts [102]. In the same study, S-HDAg was observed to enhance pol II elongation, presumably by displacing the subunit A of the negative elongation factor (NELF-A). S-HDAg was thus reported as an elongation enhancer of RNA-templated pol II transcription *in vitro* [102]. However, the observed enhancement appears to be limited to 3′-OH end additions, rather than transcription. In a subsequent study, Yamaguchi et al. reported that S-HDAg functionally interacts with pol II suggesting that S-HDAg may be involved in facilitating the uncommon RNA-directed synthesis by an RNA polymerase that is normally DNA-directed [103]. They proposed that the interaction between pol II and S-HDAg loosens what, from molecular structure studies, is considered to be a pol II clamp, thereby reducing transcriptional fidelity and allowing the recognition of the atypical RNA template.

Amidst all the reports that S-HDAg actively participates in HDV RNA transcription, there is a contradictory result showing that the presence of S-HDAg is not required for the accumulation of processed short HDV transcripts, although full-length transcripts, genomic or antigenomic, do require S-HDAg, or even L-HDAg [104]. As an explanation, it was proposed that full-length HDV RNAs are susceptible to nucleolytic degradation in the absence of S-HDAg, and, due to their size, such RNAs are more prone to be degraded than smaller RNAs. In other words, S-HDAg interacts with HDV RNAs to protect them and thereby allow their accumulation in infected cells.

Another role attributed to S-HDAg is that of HDV RNA chaperone. *In vitro* studies have reported that S-HDAg can stimulate HDV RNA ribozyme activity [105]. From such studies, it is inferred that *in vivo* S-HDAg may be directly involved in posttranscriptional processing of nascent multimeric transcripts by enhancing cleavage into unit-length molecules. It should be noted, however, that the abovementioned studies of Lazinski and Taylor indicate that, *in vivo*, HDAg is not directly needed for ribozyme cleavage and subsequent ligation [104].

S-HDAg may also be involved in deviating/redirecting other host cell components to facilitate HDV RNA replication. S-HDAg is a rather promiscuous protein in that many cellular partners have been detected.

HDV has a very small RNA genome, as mentioned earlier, and encodes only one viral protein, HDAg. Albeit the fact that a second isoform of the HDAg appears later in the replication cycle, S-HDAg and L-HDAg are not sufficient for HDV to complete its replication cycle. HDV must rely extensively on host cell factors to complete its replication cycle.

A comprehensive study using immunopurification followed by mass spectrometry identified over 100 host proteins associated with a tagged S-HDAg [105]. This set included 9 of the 12 subunits of the pol II complex, further supporting the idea that pol II is involved in HDV RNA transcription [106]. In another study, a yeast two-hybrid approach identified 30 proteins encoded by a human liver cDNA library that interacted with S-HDAg [107]. Only three proteins from this study had also been identified by the previously mentioned immunopurification approach.

How can one small protein be involved in so many interactions and perform such different functions? The answer may be related to the S-HDAg's lack of structure. Based on S-HDAg's sequence, it has been predicted that the protein is extensively disordered [108]. The prediction that S-HDAg is an intrinsically disordered protein (IDP) has been experimentally confirmed by circular dichroism measurements, which have shown that the protein has little structure apart for its coiled-coil domain [108].

In fact, S-HDAg has several characteristics generally attributed to IDPs. These proteins are commonly nucleic acid binding proteins displaying chaperone activity, such as S-HDAg. Also, a high net charge is characteristic and S-HDAg has an estimated net charge of +12 [52]. Multimerization ability is yet another feature of IDPs that is present in S-HDAg.

The lack of a rigid 3-dimensional structure may account for the protein's promiscuity and ability to take part in several interactions with distinct partners. As such, a deceivingly simple virus, encoding only one protein, can hijack the host cell mechanisms required to complete its life cycle.

## 8. Conclusion

More than 30 years after its discovery, a lot of fundamental aspects of the HDV life cycle and interaction with the host still remain unknown. But its peculiar simplicity makes all its beauty.
